# Temporal Dynamics of Cerebral Blood Flow, Cortical Damage, Apoptosis, Astrocyte–Vasculature Interaction and Astrogliosis in the Pericontusional Region after Traumatic Brain Injury

**DOI:** 10.3389/fneur.2014.00082

**Published:** 2014-06-04

**Authors:** Sonia Villapol, Kimberly R. Byrnes, Aviva J. Symes

**Affiliations:** ^1^Center for Neuroscience and Regenerative Medicine, Uniformed Services University of the Health Sciences, Bethesda, MD, USA; ^2^Department of Pharmacology, Uniformed Services University of the Health Sciences, Bethesda, MD, USA; ^3^Department of Anatomy, Physiology and Genetics, Uniformed Services University of the Health Sciences, Bethesda, MD, USA

**Keywords:** astrogliosis, cell death, cerebral blood flow, vasculature, glial scar

## Abstract

Traumatic brain injury (TBI) results in a loss of brain tissue at the moment of impact in the cerebral cortex. Subsequent secondary injury involves the release of molecular signals with dramatic consequences for the integrity of damaged tissue, leading to the evolution of a pericontusional-damaged area minutes to days after in the initial injury. The mechanisms behind the progression of tissue loss remain under investigation. In this study, we analyzed the spatial–temporal profile of blood flow, apoptotic, and astrocytic–vascular events in the cortical regions around the impact site at time points ranging from 5 h to 2 months after TBI. We performed a mild–moderate controlled cortical impact injury in young adult mice and analyzed the glial and vascular response to injury. We observed a dramatic decrease in perilesional cerebral blood flow (CBF) immediately following the cortical impact that lasted until days later. CBF finally returned to baseline levels by 30 days post-injury (dpi). The initial impact also resulted in an immediate loss of tissue and cavity formation that gradually increased in size until 3 dpi. An increase in dying cells localized in the pericontusional region and a robust astrogliosis were also observed at 3 dpi. A strong vasculature interaction with astrocytes was established at 7 dpi. Glial scar formation began at 7 dpi and seemed to be compact by 60 dpi. Altogether, these results suggest that TBI results in a progression from acute neurodegeneration that precedes astrocytic activation, reformation of the neurovascular unit to glial scar formation. Understanding the multiple processes occurring after TBI is critical to the ability to develop neuroprotective therapeutics to ameliorate the short and long-term consequences of brain injury.

## Introduction

Traumatic brain injury (TBI) can result in significant impairment of function, if the patient survives the initial impact. TBI induces a series of events in the brain that trigger an instantaneous loss of tissue and damaged area around the impact site (1). Repair of the damaged brain may result from avoiding or reducing secondary neuronal degeneration and decreasing glial activation that leads to the deterioration of the neurological state.

Excessive glutamate stimulation induces excitotoxicity predominantly in neurons and has been linked to the pathological process of various chronic CNS diseases and TBI (2). Under this pathological environment within the injured cortex, apoptosis, inflammation, gliosis, and a reduction in regional cerebral blood flow (CBF), all play a role in secondary cell injury (3). This secondary reaction is predominately located in the primary cortical lesion and around the core impact zone, referred to as the perilesional or pericontused regions (4). A collection of detrimental mechanisms contributes to this secondary injury, including edema, decreased CBF, disruption of the blood–brain barrier (BBB), necrosis, apoptosis, gliosis, excitotoxicity and energy depletion. These dynamic processes, involving glial cells and vessels, are becoming the target of potential therapeutics to treat brain trauma.

The neurovascular unit is also altered after TBI, although much less is known about this process (5). The neurovascular system is composed of a complex network of neurons, astrocytes, and cerebral blood vessels (endothelium, smooth muscle cells, and perivascular matrix) (6). Cerebrovascular dysfunction is observed after TBI with a decrease in CBF, glucose consumption, and oxygen extraction (7). However, the temporal pattern of disruption and the underlying mechanisms remain poorly understood.

Astrocytes are thought to play a crucial role in response to injury; they are important in neuronal antioxidant defense, secreting neuroprotective factors, and in maintaining the homeostasis of the extracellular environment after brain injury (8, 9). Astrocytes also provide neurons with energy from metabolic substrates and the precursors of neurotransmitters. On the other hand, astrocytes can contribute to neuronal damage by releasing glutamate in glutamate- and calcium-dependent manners and thus, support lesion progression (10–12). Further, astrocytic hypertrophy, hyperplasia, and glial scar formation have negative effects on axonal regeneration (13).

The present study reports on the patterns of neurodegeneration, astrogliosis, and neurovascular interactions from 5 h to 2 months after TBI. We examine the short and long-term relationships between vascular changes and astrocytes and their possible involvement in neuronal cell damage after adult brain injury. Collectively, our results indicate an interplay between astrocytes, blood flow, and neurodegeneration that may guide future therapeutic intervention for specific cell types at specific times after TBI.

## Materials and Methods

### Animals and controlled cortical impact injury

The experiments were performed on 75 9-week-old male C57BL/6 mice weighing 21–25 g, which were kept under 12:12 light and dark cycle with access to food and water *ad libitum*. Surgery was performed 1 week after recovery from transportation-related stress. Mice were anesthetized with isoflurane (3% induction, 2% maintained). The skull was fixed in a stereotactic frame and a 5-mm craniotomy was performed above the left parietal cortex. We performed mild–moderate controlled cortical impact (CCI) injury (coordinates: 2 mm lateral, 2 mm posterior to Bregma) at an impact depth of 1 mm, with a 2 mm diameter round impact tip (speed 3.6 m/s, dwell time 100 ms) and a 12° angle, using an electromagnetically driven CCI injury device (Impact One stereotaxic impactor CCI, Leica Microsystems Gmbh, Wetzlar, Germany). The bone flap was replaced but not sealed, the skin was sutured, and the mice were allowed to recover fully from anesthesia before transfer to their home cages. The mice were sacrificed at 5 h, 1, 3, 7, 14, 30, and 60 days after CCI injury. The control group for all comparisons was comprised of age-matched uninjured naïve mice (*n* = 4–5). All animal studies were approved by the USUHS Institutional Animal Care and Use Committee and were conducted in accordance with the NRC guide to the Care and Use of Laboratory Animals.

### Determination of cerebral blood flow

Cerebral blood flow was measured in the pericontusional region using a laser-Doppler flowmeter (PeriFlux System 5000 LDPM, Perimed). Changes in CBF were taken using a flexible fiber optic extension to the LDPM probe tip 404 as described previously (14). Baseline values were recorded after positioning the fiber optic extension on the skull at the −2 mm posterior, and 2 mm lateral from Bregma. After CCI injury, to record CBF, animals were anesthetized with isoflurane, the fiber optic extension was positioned on the skull around the craniotomy site, 5–10 measurements were taken at each time point for each animal and averaged. CBF was recorded starting at 15 s after cortical impact, and subsequently at 2 h, and 1, 3, and 30 days after CCI injury. Changes in CBF were expressed as the percentage of the baseline value recorded before CCI injury.

### Nissl staining and lesion volume measurements

Mice were sacrificed at 5 h, 1, 3, 7, 14, 30, and 60 days post-injury (dpi) by transcardial perfusion with 4% paraformaldehyde (PFA) in phosphate buffer. Brains were removed and placed in 4% PFA overnight, then transferred to 30% sucrose solution and stored at 4°C. Brains were cut in 30 μm-thick sections using a microtome and were stored in cryoprotectant solution. Every third section was chosen for Nissl staining to reveal histology of the cortical lesion area. Brain slices were mounted on polylysine-coated slides and stained for 20 min with 0.1% cresyl-violet (Sigma) dissolved in distilled water and filtered. Slides stained were dehydrated for 2 min using 100, 95, 70, and 50% ethanol, cleared in xylene for another 2 min, covered with DPX, and coverslipped. Lesion volume was obtained by multiplying the sum of the lesion areas by the distance between 9 and 15 brain sections. Percent lesion volume was calculated by dividing each lesion volume by the total ipsilateral hemisphere volume (similarly obtained by multiplying the sum of the areas of the ipsilateral hemispheres by the distance between sections).

### Immunofluorescence analysis

Sections were blocked with 10% normal goat serum (NGS) in PBS with 0.1% Triton X-100 (PBS-T) for 1 h. The following primary antibodies were incubated at 4°C overnight in PBS-T and 5% NGS: anti-glial fibrillary acidic protein (GFAP), either mouse monoclonal (1:2000, Millipore) or chicken polyclonal (1:400, abcam) for astrocytes; anti-vimentin, mouse monoclonal (1:200, Sigma) for reactive astrocytes; anti-NeuN, mouse monoclonal (1:200, Chemicon) for mature neurons; anti-Iba-1 rabbit polyclonal (1:750, Wako) for microglia; and anti-collagen IV rabbit polyclonal (1:3000, Chemicon) a component of the basal lamina that is used as a specific marker for cerebral microvessels (15). Sections were washed in PBS-T three times and incubated with the corresponding Alexa Fluor 568-conjugated (red) and Alexa Fluor 488-conjugated (green) IgG secondary antibodies (all 1:1000, Invitrogen) for 2 h at room temperature. Sections were rinsed with PBS and distilled water and coverslipped with ProLong Gold antifade reagent with DAPI (Invitrogen).

### Cell death assay

Sections were processed for DNA strand breaks (TUNEL assay, labeling of fragmented DNA) using the Fluorescence *In situ* Cell Death Detection Kit (Roche, IL, USA), according to the manufacturer’s instructions. TUNEL-positive nuclei were counted in cortical regions in three to five coronal sections for each animal, with five animals per group.

### Quantitative and densitometry analysis

Quantitative image analysis of the immunoreactive areas for GFAP, Iba-1, and collagen IV clusters positive cells were performed on five cortical sections per brain through the level of impact site (AP: 2.0 mm) taken with the ×20 objective and using the same densitometric analysis method as previously described (16). Immunofluorescence intensity was calculated using the threshold method and defined as the number of pixels, divided by the total area (square millimeter) in the imaged field with the average background subtracted. The grade of astrogliosis was calculated by GFAP immunoreactivity values (IR-GFAP) multiplied by the number of GFAP-positive astrocytes. To assess astrocytic interaction with microvessels, we co-stained brain sections with GFAP and collagen IV; colocalization clusters were determined by pixel-by-pixel quantification of both markers that connected astrocytes and vessels. Images were acquired on an Olympus BX61 with attached qImaging Retiga EXi Aqua CCD camera, and iVision software (BioVision Technologies, Exton, PA, USA). For colocalized images, double-stained cells were analyzed with a Zeiss confocal-laser scanning microscope (LSM 510) equipped with argon and He/Ne 488 and 568 nm laser. Images were taken at 20×, 40×, and 63× magnification, and cropped and adjusted using Adobe Photoshop CS5.

### Statistical analyses

Data were analyzed using one-way analysis of variance (ANOVA) for comparison of measurements at different time points after CCI injury with those of naïve control brains. Quantitative data for all figures and tables are expressed as mean ± SEM, except for CBF measurements that are expressed as mean ± SD. All statistics were analyzed using Prism software (Graphpad).

## Results

In this study we determined, in the pericontusional cortical region, the temporal progression of post-injury alterations in CBF, cell death, and the vascular–astroglial response in the mouse after mild–moderate CCI injury. The study of these perilesional phenomena is essential to understanding the metabolic equilibrium between glial cells, vasculature, and dying cells. We chose to use naïve mice as our controls rather than those with craniotomy as we have previously shown with an identical injury, the mice subject to craniotomy had equivalent numbers of GFAP-positive proliferative astrocytes as those undergoing CCI (17). Our experience is consistent with that of others who have shown that craniotomy alone is equivalent to a minor injury in terms of the acute inflammatory response (18). As we wanted to compare injured mice with uninjured, we used naïve mice as controls in all our experiments.

### Decrease of cerebral blood flow in the pericontusional area after CCI injury

**Figure 1:**
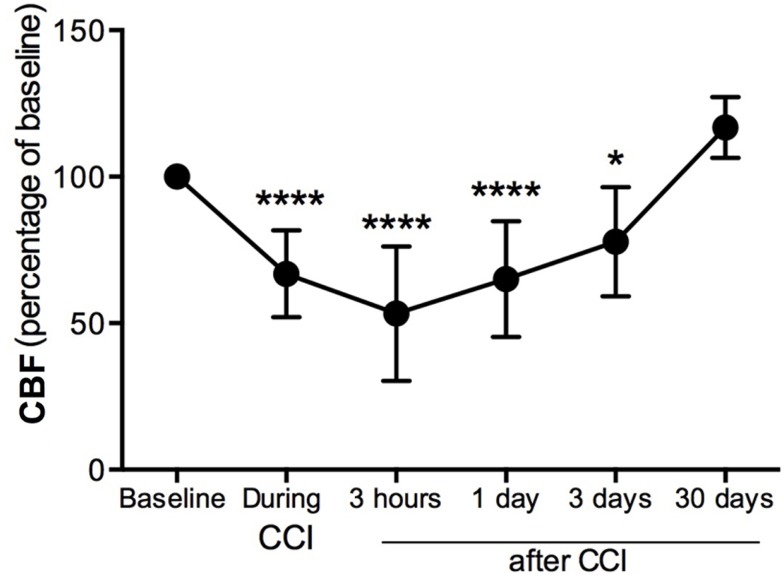
**Cerebral blood flow decreases in the pericontusional region after CCI injury**. Cerebral blood flow (CBF) measurements were expressed as percentage of baseline levels before injury. CBF was reduced in the injured cerebral cortex during CCI injury, and at 3 h, 1 dpi, and 3 dpi; it increased at 4 weeks after CCI injury. Values are expressed as the mean ± SD, *n* = 8–10 per group, *p < 0.05, ***p < 0.0001 compared to baseline.

Cerebral blood flow was measured in the pericontusional region around the impact site where a hole was perforated in the skull. CBF was decreased 34% immediately (seconds) after the impact contusion (“during CCI”) (Figure 1) compared to baseline levels. Three hours after injury, we found the CBF to be 53% of baseline levels, the lowest CBF measured. CBF increased at 1 and 3 dpi, reaching 35 and 23%, respectively, of the baseline levels initially obtained before CCI injury (Figure 1). By 30 dpi, CBF was restored to baseline.

### Pericontusional-damaged areas and cortical cavity generated after CCI injury

**Figure 2:**
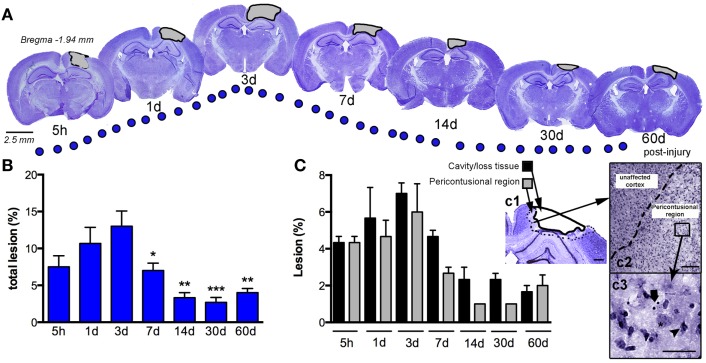
**Temporal progression of the cortical lesion**. **(A)** Representative drawings of Nissl staining from coronal sections at 5 h, and 1, 3, 7, 14, 30, and 60 days post-CCI injury (dpi). Black lines delimit the lesioned cortex (gray). **(B)** Graph shows the percent lesioned area relative to the area of the whole ipsilateral hemisphere, starting at 5 h after injury, with a peak at 3 days and remaining up to 60 days after injury. **(C)** Graph illustrates the composition of the total lesion that is either cavity (or lost tissue) versus the damaged pericontusional region as a percentage of the area of the total ipsilateral hemisphere at different times post-injury. (c1) Representative images showing the delimited damaged cortical regions corresponding to cavity or tissue loss or the pericontusional region. High magnification image of the unaffected cortex and pericontusional region (c2) containing apoptotic cells (c3, arrow) and chromatin condensate (c3, arrowhead). Scale bar; 100 μm (c1), 50 μm (c2), and 20 μm (c3). Values are expressed as the mean ± SEM, *n* = 3–5 per group, *p < 0.05, **p < 0.01, ***p < 0.0001 compared to 3 dpi.

We assessed the pattern of cortical lesion volume at 5 h and 1, 3, 7, 14, 30, and 60 dpi (Figure 2), dividing tissue damage into lesion cavity and pericontusional area (Figure 2C). Nissl staining revealed an immediate loss of cortical tissue after the impact-rounded tip entered the sensorimotor cortex. As early as 5 h post-injury, neocortical Nissl staining diminished in intensity, and scattered cell loss and shrinkage was evident through all neocortical layers (Figure 2). A damaged region around the contusion site was also generated early. Damaged tissue was evident via a loss of Nissl intensity with the pyknotic and apoptotic neurons delimiting the pericontusional region (Figures 2c1–c3). At 1 dpi, the injury cavity increased despite a loss of necrotic tissue, while the pericontusional region remained unchanged. At 3 dpi, the cavity expanded further, contributing to the peak volume of both lesioned cortex and damaged pericontusional regions (Figures 2B,C). This cortical lesion volume decreased at 7, 14, 30, and 60 dpi.

### Biphasic peak of cell death in the pericontusional region

**Figure 3:**
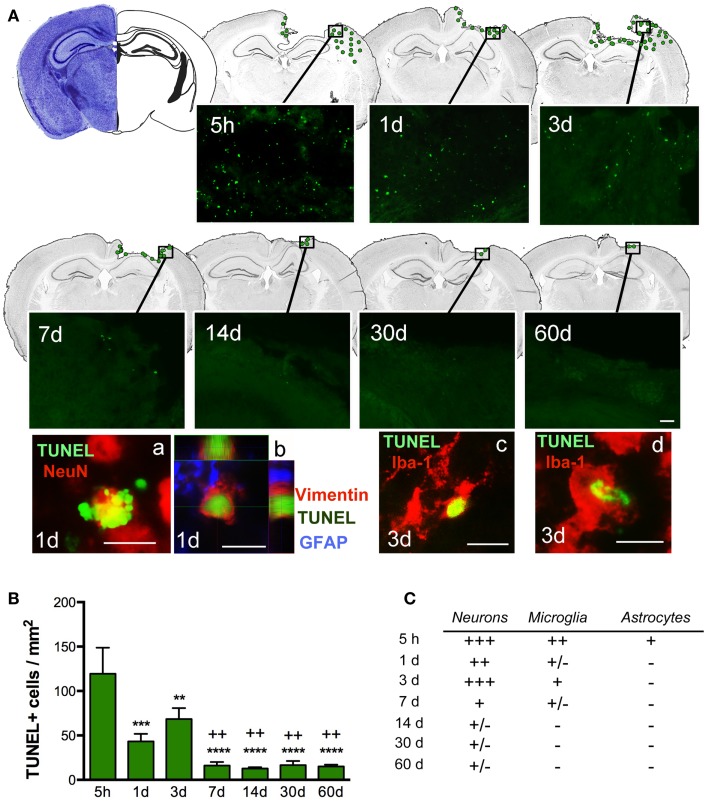
**Distribution of apoptotic cells in the perilesional cortex at several time points after TBI**. **(A)** Illustration of the spatial–temporal distribution of TUNEL-positive cells (green dots). TUNEL-positive cells were distributed around the impact site at 5 h until 3 days post-injury (dpi) but were then limited to the lesion border at 7, 14, 30, and 60 dpi. Images of TUNEL-positive cells in a single field for each time point are shown. High power images indicate that dying cells (TUNEL, green), are neurons (NeuN, red) (a) reactive astrocytes (vimentin, red, and GFAP, blue) (b) or microglia (Iba-1, red) with nuclear TUNEL staining (c). Phagocytic microglia/macrophages (d) colocalize with TUNEL while engulfing TUNEL-positive cells. Scale bar; 20 μm (a–d) and 50 μm **(A)**. **(B)** Plot depicts the temporal density of TUNEL-positive cells in the injured cortex with an early peak at 5 h after injury and declining by 7 dpi. **(C)** Table highlighting the identity of TUNEL-positive cells at different time points. Degree of colocalization of TUNEL-positive cells with neurons (NeuN), microglia (Iba-1), and astrocytes (GFAP) is graded as; +++ (high), ++ (moderate), + (occasional), ± (in rare cases), and − (no observed) at several time points after CCI injury. Values are expressed as mean ± SEM, *n* = 3–5 per group, ***p < 0.001, **p < 0.01, ***p < 0.0001 compared to 5 h group ^++^*p* < 0.01 compared to 3 dpi group.

**Figure 4:**
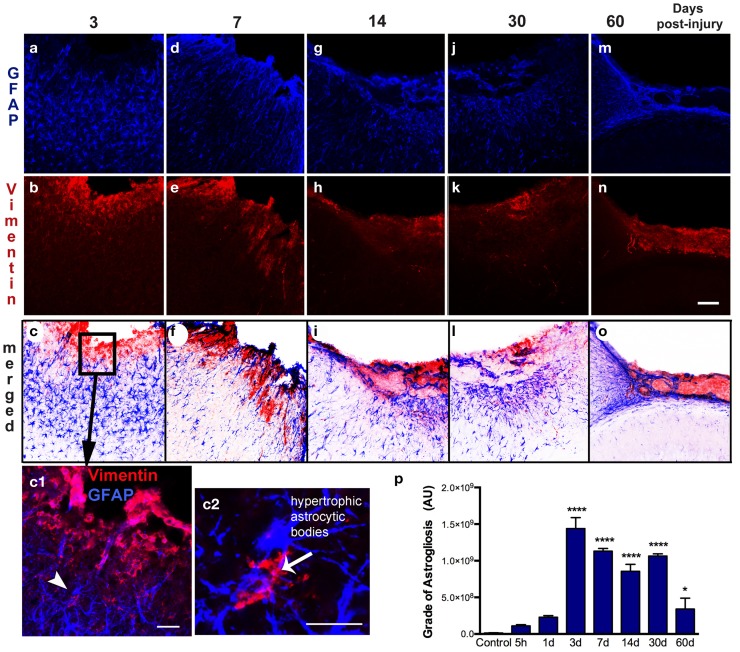
**Spatial–temporal pattern of astrogliosis**. GFAP (a–m) and vimentin (b–n) immunoreactivity in the ipsilateral cortex of lesioned mice at 3, 7, 14, 30, and 60 days post-injury (dpi). The sensorimotor cortex lesion of the young adult mouse brain evoked extensive reactive astrogliosis at 3 dpi, as shown by upregulation of GFAP and vimentin expression, surrounding the lesion site (a–c). Enlarged detail of the lesion site (box in c) shows that reactive astrocytes are vimentin-positive (arrowhead in c1) have large hypertrophic astrocytic bodies and an increased number of short processes (arrow in c2). These reactive astrocytes have an enlarged body with an increased number of short, thick processes that are directed towards the border of the lesion at 7 dpi (d–f). Extensive reactive astrogliosis was primarily detected in the border of the damaged cortex at 14 dpi (g–i). Once the glial scar has formed, both GFAP and vimentin expression is weak and restricted to the superficial part of the injured cortex at 30 and 60 dpi (m–o). Quantification of the grade of astrogliosis (p) showed a peak at 3 dpi that decreases overtime. Scale bar; 100 μm (a–o), 50 μm (c1), and 20 μm (c2). Values are expressed as mean ± SEM, *n* = 3–5 per group, ****p < 0.0001, *p < 0.05 compared to control.

Quantification of cell death (by TUNEL-positive cells) within the ipsilateral sensorimotor cortex following injury revealed marked loss overtime during the evolution of cortical damage. By 5 h after injury, we observed a significant increase in the number of apoptotic cells that were diffusely distributed throughout the pericontusional region (Figure 3A). At 1 dpi, there was a reduction in dying cells; however, at 3 dpi there was a secondary peak in apoptosis around the lesioned area, which correlated with increased astroglial reactivity (Figure 4). TUNEL labeled morphologically distinct cells were principally neurons with apoptotic bodies and chromatin condensation (Figure 3Aa). Dying astrocytes were also identified at 1 and 3 dpi with double staining via vimentin and GFAP (Figure 3Ab). Additionally, Iba-1-positive microglial cells with TUNEL-positive nuclei (Figure 3Ac) showing typical phagocytic morphology were observed at 3 dpi (Figure 3Ad). At 7 dpi, we occasionally observed dying neurons and sporadic Iba-1/TUNEL-positive cells. At longer time points (14, 30, and 60 dpi), rare cases of dying neurons were observed (Figure 3C). Combined, these findings suggest that neurons are the main cell population susceptible to death, especially at early time points after TBI.

### Dynamic of astrocytic activation in response to cortical damaged

Astrogliosis, observed by GFAP staining, was not apparent at early time points (5 h post-injury and 1 dpi) but appeared in the pericontusional region at 3 dpi (Figure 4). Activated astrocytes possessed thick, labeled processes, and hypertrophic astrocytic bodies (Figure 4c2). Reactive astrocytes occupied an extended region corresponding to cortical layers II through VI at 3 dpi, mainly around the impact site and near the lesion border. GFAP/vimentin double-labeled cells identified astrocytic activation that was extended to the peripheral cortical regions near the lesion core. At 3 dpi, vimentin expressing astrocytes were distributed around the impact site, mainly associated with reactive astrocytes in the gliotic tissue (Figures 4a–c). At 7 dpi, the branches of vimentin/GFAP-positive cells were arranged parallel to each other and perpendicularly to the border of the lesion starting to form the glial scar (Figures 4d–f). GFAP-positive astrocytes were observed at 14 dpi bordering the lesion near the formation of a glial scar (Figures 4g–i). This chronic astrogliosis persisted up until 60 dpi with a notable involvement in maintaining the glial scar, defining the border of the cavity (Figures 4m–o). Quantitative analysis revealed that the grade of astrogliosis had a peak at 3 dpi (Figure 4p). No changes in GFAP upregulation in undamaged areas of the contralateral or naïve control cortex were observed (data not shown).

### Astrocytic–vasculature interaction after injury

**Figure 5:**
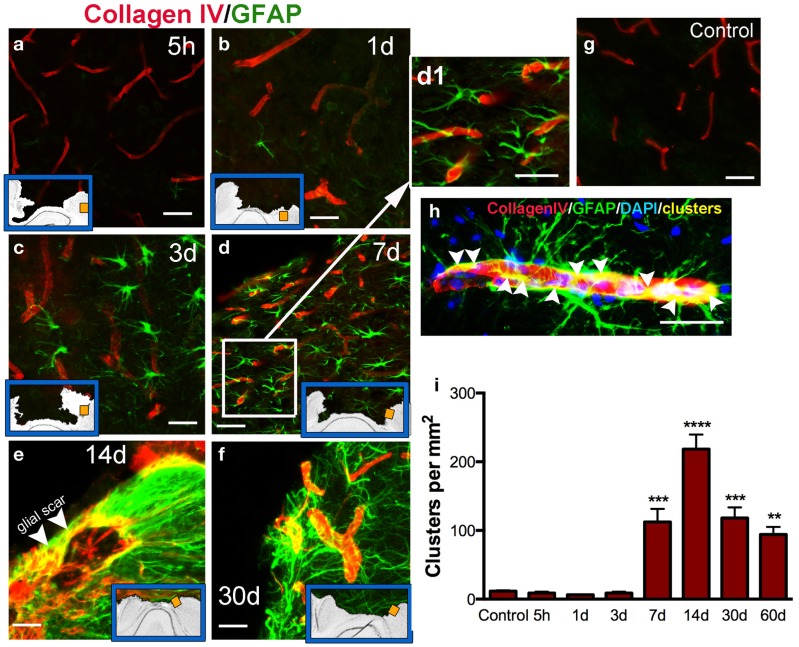
**Vascular interaction with astrocytes**. Representative photomicrographs showing double immunohistochemistry for microvessels stained using Collagen type IV (red) and GFAP (astrocytes, green) in the cortex, at 5 h (a), 1 day (b), 3 days (c), 7 days (d, d1), 14 days (e,h), and 30 days (f) post-injury (dpi), and in uninjured mice (control, g). Inset blue boxes indicate the location within the cortical regions that the image was taken. (h) Shows the interaction of blood vessels and astrocytes. Integration of their respective stains results in yellow colocalization (called clusters). (i) Density of clusters (measured in pixels per area) in the pericontusional regions of the cortex. Clusters are first detected at 7 dpi, and peak at 14 dpi. Values are expressed as mean ± SEM, *n* = 3–5 per group, **p < 0.01, ***p < 0.001, ***p < 0.0001 compared to control levels.

Cerebral vasculature within the pericontusional region was stained with the collagen IV antibody; we observed varying distribution of immunoreactivity after injury (Figures 5a–f). Distribution of cortical microvessels in the perilesional region at 5 h post-injury (Figure 5a) and 1 dpi (Figure 5b) was similar to uninjured control brains (Figure 5g). After 3 dpi, vessels increased in thickness (Figures 5c–f). Interactions between astrocytes and vessels were detectable at 7 dpi, and this interaction peaked at 14 dpi (Figure 5e). Initial formation of the glial scar was detected at 7 dpi (Figures 5d,d1). Interactions between astrocytes and thick-walled blood vessels remained detectable until 30 dpi (Figures 5f,h). Astrocytic-vessel contacts with the cavity border were maintained until 60 dpi (Figure 5i).

**Figure 6:**
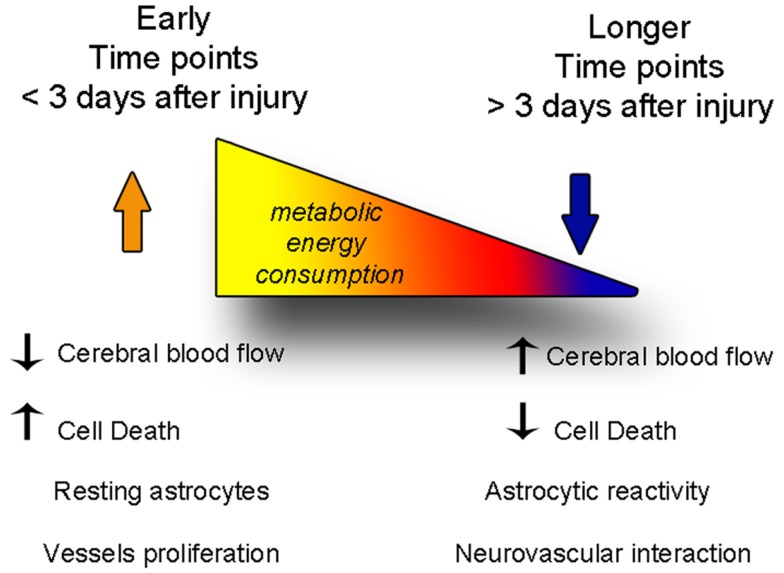
**The balance between degenerative and vascular phenomena is altered with time after injury**. At early time points, cell death and decreased cerebral blood flow predominate, while astrocytes are not yet reactive. At later time points, cerebral blood flow levels are restored fewer cells die, but astrogliosis becomes stronger, and the neurovascular units reform.

## Discussion

In this study, we have characterized the pattern of neurodegeneration and astrogliosis occurring in the cortex, in conjunction with glial–vascular interactions and alterations of regional CBF, at multiple times points after CCI injury. Our regional analysis of neurovascular interaction provides further understanding of the different responses of astrocytes and vasculature after brain injury.

Our results suggest that a chronically progressive degenerative process in the pericontusional-injured region is initiated hours after CCI injury, but persists for weeks after impact. Thus, there seems to be a relatively broad therapeutic window for drug administration to exert maximum efficacy and ameliorate the short and long-term consequences of TBI. The cascade of events that lead to the initial response could correspond with the rapid release of glutamate and excitotoxic metabolites that subsequently induce cell death (19). This phenomenon has been observed in several models of brain injury, mainly after ischemia, where necrotic cell death has been observed to have a quick yet extended response in the ipsilateral hemisphere (20). We have shown here that a cortical cavity, generated via an impact tip, had maximal extension at 3 days after CCI injury. However, other studies in different brain injury models have shown that the size of the cortical cavity was fully developed as early as 6 h after TBI (21). At 3 dpi, we identified apoptotic cell death located in the lesion periphery, limited to the lesion border and mainly corresponding to neuronal death. This results from neurons being more susceptible to CNS insults than astrocytes, as they have limited antioxidant capacity and rely on their metabolic coupling with astrocytes to combat oxidative stress (20, 22, 23).

Astrocytes become reactive after trauma, ischemia, or neurodegenerative diseases (astrogliosis). Hypertrophy of astrocytic processes is accompanied by the upregulation of GFAP and vimentin, two intermediate filaments that are abundantly expressed in immature and reactive astrocytes (24, 25). Reactive astrocytes can form GFAP positive filaments in vimentin-deficient mice, but with more compact bundles than in wild-type astrocytes, showing that both vimentin and GFAP normally contribute to the cytoskeletal structure of astrocytes (26). Other studies have suggested that GFAP-positive reactive astrocytes contribute to the resistance of CNS tissue to specific types of severe mechanical stress (27), taking up excess glutamate (10), rebuilding the blood–brain barrier (28, 29), and production of growth factors (30).

Interestingly, we found that the pericontusional region at early time points after injury is devoid of GFAP-positive astrocytes. Further, we found that some astrocytes are TUNEL-positive 5 h after injury (Figure 3) illustrating that astrocytes are vulnerable in the acute phase of injury. This early astrocytic demise has been previously demonstrated in ischemic animal models (31). Previous studies have also suggested that there is an immense variability in the subpopulation structure of astrocytes, characterized by several grades of susceptibility in their response to brain injury (25, 32). We show that, in the vicinity of the lesion, reactive astrocytes on day 3 increase the thickness of their main cellular processes and convert to a hypertrophic morphology. We also identified a strong astrogliosis from 7 days after injury until 2 months after injury, where astrocytes contribute to the formation of the glial scar.

Astrocytic hypertrophy, hyperplasia, and glial scar formation all have negative effects on regeneration, although some evidence favors a positive role for astrocytes in brain injury as they have phagocytic capabilities and are partially responsible for clean up of the lesion site in the acute stages after trauma (33). While, at later stages they facilitate the formation of a post-traumatic glial scar (25, 34). Formation of the glial scar, a barrier composed of extracellular matrix, where collagen IV is a major constituent of basement membranes, has been considered a major factor involved in inhibition of neurite outgrowth and repair after CNS injuries (15). Thus, some treatments under development seek to limit the formation of the astroglial scar in order to repair the injured CNS. Some studies have shown how the inhibition of collagen IV synthesis enhances regeneration of axons that become remyelinated with compact myelin after brain injury (35, 36). Spatial–temporal analysis of reactive astrocytes in the injured cortex may help to provide a better understanding on the role of diverse astrocytes, and accordingly, the distinct glial responses, depending on the distance from the injury core and the time point studied.

Astrocytes, as a result of their close relationships with neurons, microglial cells, and blood vessels have long been hypothesized to be involved in cerebrovascular regulation (1, 37). The terminal processes or “endfeet” of astrocytes cover the majority of the albuminal vascular surface of microvessels, intracerebral arterioles, and venules (38). Glutamate released during synaptic transmission stimulates astrocytic calcium signaling, which in turn induces vasodilatation (39). Other agents released from neurons or vessels participate in the increase in CBF induced by neural activity, such as nitric oxide, ATP, or calcium (40–42). While the interaction between adjacent reactive astrocytes and vasculature in the pericontusional-injured cortex remains minimal at early stages, it becomes densely packed near the lesion borders at later times after trauma. Our immunohistological analysis highlighted the link between astrocytes and large vessels in the border of the lesion starting at 7 days after injury when the surfaces of large to medium-size vessels were densely covered by GFAP astrocytic endfeet (Figure 5).

The structural and functional integrity of the brain depends on a continuous vascular supply of oxygen and glucose, and if CBF is interrupted or unable to meet an increased metabolic demand, neurons cease to function, and reduced thresholds for activation of pathways leading to delayed neuronal death (1, 8, 43). A phasic elevation in CBF after acute head injury is a necessary condition for achieving functional recovery (44). Lower levels of blood flow further contribute to an excitotoxic cascade explosion with the release of glutamate to the extracellular space, as well as other toxic metabolites that induce a rapid expansion of cell death surrounded by an intense astrocytic reaction (33, 45). Mean arterial pressure (MAP), intracranial pressure, and other physiological variables also influence CBF (46).

Cerebral blood flow has been measured following experimental TBI (41, 47, 48) and an increase in CBF has a neuroprotective role after brain injury (8). Our results demonstrate a sudden decrease in CBF after traumatic impact within the cerebral cortex, from the first seconds after the impact tip was removed from the brain, and persisting for several days. Coinciding with a decrease of CBF in the pericontusional cortical regions, a massive astrocytic response, cell death, and an increase in perilesional vasculature all occur after mild–moderate TBI in mice. Astrocytes have processes in direct contact with blood vessels, which has long indicated that they may be involved in neurovascular regulation. These cells have the ability to dilate and constrict blood vessels and finely modulate the distribution of CBF changes during neuronal activation (49, 50) and energy metabolism (7). We show that CBF was restored to baseline values by 30 dpi (Figure 1) but did not determine at which point between 3 and 30 dpi, the CBF normalized. However, we also show increased formation of astrocytic–vascular clusters starting at 7 dpi and continuing until 60 dpi. Thus, it is possible that the formation of these clusters could help restore the CBF to baseline levels, even though these structures are not found in naïve mouse brain.

The pathological disruption of vessel interactions with astrocytes may be involved in neurovascular regulation, glial scar formation, and CBF. However, despite intense research in neurovascular interactions, the role that astrocyte–vasculature interaction may play in neuronal survival remains poorly understood. Modulating the energy demands or interacting with microvessels to influence the vascular flow in the pericontusional cortex may be one mechanism by which astrocytes contribute to neuronal recovery.

## Conclusion

In this study, we determined the spatial–temporal course of the astrocytic–vascular reaction, in parallel with apoptotic cell death, after mild–moderate brain injury in mice. We focused on assessing changes detected in the pericontusional cortical regions, targeting the possible involvement of astrocytes with cerebrovascular dysfunction and neurodegeneration.

A clear understanding of the molecular regulation of cellular damage including the neurovascular unit will be crucial for the design of new treatments for brain injury. Further elucidation of the temporal response of the astroglial–vasculature complex after brain injury should indicate potential critical points for intervention to increase CBF after injury that should have clinical relevance.

## Conflict of Interest Statement

The authors declare that the research was conducted in the absence of any commercial or financial relationships that could be construed as a potential conflict of interest.
